# OX40 expression enhances the prognostic significance of CD8 positive lymphocyte infiltration in colorectal cancer

**DOI:** 10.18632/oncotarget.5940

**Published:** 2015-09-29

**Authors:** Benjamin Weixler, Eleonora Cremonesi, Roberto Sorge, Manuele Giuseppe Muraro, Tarik Delko, Christian A. Nebiker, Silvio Däster, Valeria Governa, Francesca Amicarella, Savas D. Soysal, Christoph Kettelhack, Urs W. von Holzen, Serenella Eppenberger-Castori, Giulio C. Spagnoli, Daniel Oertli, Giandomenica Iezzi, Luigi Terracciano, Luigi Tornillo, Giuseppe Sconocchia, Raoul A. Droeser

**Affiliations:** ^1^ Department of Surgery, University Hospital Basel, Basel, Switzerland; ^2^ Institute of Surgical Research and Hospital Management (ICFS) and Department of Biomedicine, University Hospital Basel, Basel, Switzerland; ^3^ Department of Systems Medicine, University of Rome “Tor Vergata”, Rome, Italy; ^4^ IU Health Goshen Center for Cancer Care, Goshen, IN, USA; ^5^ Institute of Pathology, University Hospital Basel, Basel, Switzerland; ^6^ Institute of Translational Pharmacology, National Research Council, Rome, Italy

**Keywords:** OX40, CD8, colorectal cancer, prognosis, microenvironment

## Abstract

**Background:**

OX40 is a TNF receptor family member expressed by activated T cells. Its triggering by OX40 ligand promotes lymphocyte survival and memory generation. Anti-OX40 agonistic monoclonal antibodies (mAb) are currently being tested in cancer immunotherapy. We explored the prognostic significance of tumor infiltration by OX40+ cells in a large colorectal cancer (CRC) collective.

**Methods:**

OX40 gene expression was analyzed in 50 freshly excised CRC and corresponding healthy mucosa by qRT-PCR. A tissue microarray including 657 clinically annotated CRC specimens was stained with anti-OX40, -CD8 and -FOXP3 mAbs by standard immunohistochemistry. The CRC cohort was randomly split into training and validation sets. Correlations between CRC infiltration by OX40+ cells alone, or in combination with CD8+ or FOXP3+ cells, and clinical-pathological data and overall survival were comparatively evaluated.

**Results:**

OX40 gene expression in CRC significantly correlated with FOXP3 and CD8 gene expression. High CRC infiltration by OX40+ cells was significantly associated with favorable prognosis in training and validation sets in univariate, but not multivariate, Cox regression analysis. CRC with OX40^high^/CD8^high^ infiltration were characterized by significantly prolonged overall survival, as compared to tumors with OX40^low^/CD8^high^, OX40^high^/CD8^low^ or OX40^low^/CD8^low^ infiltration in both uni- and multivariate analysis. In contrast, prognostic significance of OX40+ and FOXP3+ cell infiltration was not enhanced by a combined evaluation. Irrespective of TNM stage, CRC with OX40^high^/CD8^high^ density infiltrates showed an overall survival similar to that of all stage I CRC included in the study.

**Conclusions:**

OX40^high^/CD8^high^ density tumor infiltration represents an independent, favorable, prognostic marker in CRC with an overall survival similar to stage I cancers.

## INTRODUCTION

Colorectal cancer (CRC) represents the second most common cause of cancer-related death [1]. Surgical resection remains the mainstay of CRC therapy, and complete removal of the tumor may be achieved in a majority of patients. Radiotherapy, and chemotherapy represent additional standard treatments, currently administered according to histological findings, using the TNM staging system [2].

Histological staging however, fails to account for recurrences observed in patients treated for early stage CRC or for long term survival of patients bearing advanced stage CRC [3]. A variety of reports convincingly indicate that the composition of the tumor microenvironment is critical for CRC progression and that the immune system plays a pivotal prognostic role [4-6].

High densities of infiltrating CD8+ T cells are associated with improved disease-free and overall survival in CRC [4-8] and the analysis of tumor infiltration by immune cells has been suggested to outperform the prognostic significance of TMA staging [5, 6]. Molecular mechanisms underlying the anti-tumor effects of the immune infiltrate are largely unclear [4, 9]. Nevertheless, the expression of activation markers by CRC infiltrating CD8+ T cells has been shown to improve their predictive potential [8]. Unexpectedly, CRC infiltration by FOXP3+ regulatory T cells (Treg) and myeloid cells was also found to be associated with improved prognosis [10-12], at difference with a variety of cancers of different histological origin [13, 14].

OX40 (CD134) is a co-stimulatory, trans-membrane molecule of the tumor necrosis factor-receptor superfamily [15, 16] expressed by activated CD4+ and CD8+ T cells [17-19]. Engagement of OX40 by OX40-ligand expressed by antigen presenting cells (APC) enhances CD4+ and CD8+ cell proliferation, stimulates cytokine production and promotes survival of antigen-specific memory T cells [20-22]*.* Based on this background, OX40 targeted immunotherapy treatments are being tested in patients with advanced cancers [23, 24].

A previous study, based on the analysis of 72 patients with CRC, suggests that OX40 expression by CRC infiltrating cells correlates with favorable prognosis [25]. This information could be of potentially high clinical relevance since it might contribute to the definition of a constellation of markers allowing a more precise identification of patients with CRC who might benefit from current therapies, while sparing unnecessary treatment to others. Furthermore, patients potentially taking advantage of OX40 targeted immunotherapy might also be characterized.

We used a tumor microarray (TMA) including >600 clinically annotated CRC to address the prognostic significance of CRC infiltration by OX40+ cells, as evaluated in combination with CD8+ and FOXP3+ cell infiltration.

## RESULTS

### OX40 gene expression in CRC and healthy mucosa

**Figure 1:**
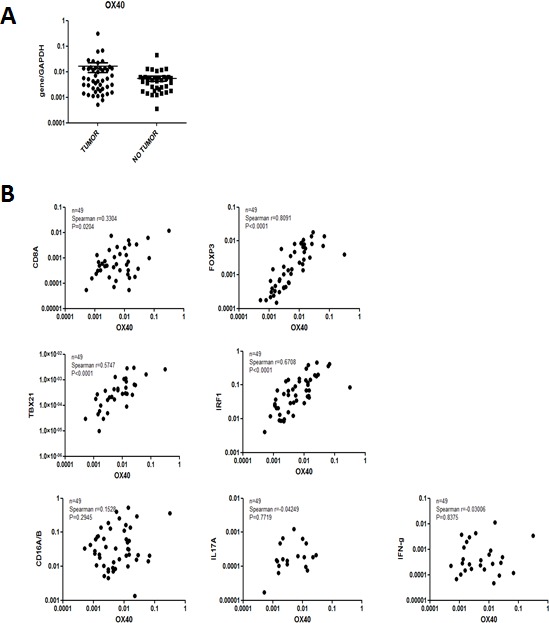
Gene expression profiles in CRC Total cellular RNA was extracted from freshly excised CRC tissues (*n* = 48) and corresponding healthy mucosa sampled at distance from the tumor and reverse transcribed. Specific gene expression was analyzed by qRT-PCR, using, as reference, GAPDH house-keeping gene expression.

We comparatively addressed OX40 gene expression in CRC tissues and in corresponding healthy mucosa sampled at distance from the tumor (*n* = 49). We found (Figure 1A) that OX40 gene is expressed to similar extents (*P* = 0.3) in cancerous and healthy colon tissues. These results were matched by publicly available data indicating that in five out of seven databases OX40 gene expression did not significantly differ in CRC and healthy tissues [29, 32] (data not shown),

### Expression of OX40 gene and genes associated with favorable clinical course in CRC

Expression of a variety of immune cell markers within CRC tissues has been shown to be associated with defined clinical outcomes [4-6]. Based on this background, we assessed the correlation between OX40 gene expression and that of a panel of genes of known prognostic significance.

In our cohort of CRC tissues (*n* = 49) OX40 gene expression was very strongly (Spearman r = 0.8, *P* < 0.0001) associated with FOXP3 [10, 11] gene expression (Figure 1B). Strong associations were also evident between OX40 and IRF1 (Spearman r = 0.67, *P* < 0.0001) or TBET (Spearman r = 0.57, *P* < 0.0001) genes [5, 6]. Furthermore, OX40 gene expression was also moderately associated with CD8 gene expression (Spearman r = 0.33, *P* < 0.02). In contrast, no significant associations were detected between OX40 and CD16, IL17A or IFN-γ gene expression [12, 37] (Figure 1B). These data were largely consistent with those emerging from TCGA database (Supplementary Figure 1), although in this cohort (*n* = 380) OX40 gene expression was also significantly associated with CD16 and IFN-γ gene expression. Taken together these data urged the evaluation of the prognostic significance of OX40+ infiltrate in CRC.

### TMA analysis

A total of 657 CRC tissues were analyzed. Median age was 71 years (range: 36-96), 54.6% of the patients were female, and 45.4% were male. 65% of the tumors were located in the left hemicolon, and the remaining 35% in the right hemicolon. Median tumor size was 50mm (range: 5-170). Most patients presented T3 lesions (63.3%), and 50.7% were node negative (N0). 85.5% were grade 2 tumors and 61.3% showed infiltrative tumor border configuration. Vascular invasion was absent in the majority of cases (70%). TMA included 552 MMR-proficient tumors and 105 MMR-deficient tumors (16%), as defined according to MLH1, MSH2 and MSH6 expression [38]. Median overall survival was of 92 months (0-152) (Table 1). This collective was randomly splitted into two similar training and validation subsets (Table 1s).

**Table 1:** Clinical-pathological characteristics of the overall CRC patient cohort and their association with levels of OX40+ infiltrate

	Total N=657*		OX40 ^low^ N=440*		OX40 ^high^ N=217*		OX40 low vs high
Characteristics	N or mean	(% or range)	N or mean	(% or range)	N or mean	(% or range)	P**
							
Age, years (median, mean)	71, 69.9	(36-96)	72, 70.5	(36-96)	70, 68.6	(40-90)	0.02
							
Tumor size in mm (median, mean)	50, 51.7	(5-170)	45, 51.5	(5-170)	50, 50.2	(7-160)	0.827
							
Sex							0.539
Female (%)	359	(54.6)	247	(56.1)	112	(51.6)	
Male (%)	298	(45.4)	193	(43.9)	105	(48.4)	
							
Anatomic site of the tumor							0.037
Left-sided (%)	425	(64.7)	272	(61.8)	153	(70.5)	
Right-sided (%)	228	(34.7)	165	(37.5)	63	(29.0)	
							
T stage							
T1 (%)	29	(4.4)	11	(2.5)	18	(8.3)	<0.0001
T2 (%)	103	(15.7)	62	(14.1)	41	(18.9)	
T3 (%)	416	(63.3)	284	(64.5)	132	(60.8)	
T4 (%)	91	(13.8)	74	(16.8)	17	(7.8)	
							
N stage							
N0 (%)	333	(50.7)	203	(46.1)	130	(59.9)	0.002
N1 (%)	174	(26.5)	127	(28.9)	47	(21.7)	
N2 (%)	128	(19.5)	97	(22.0)	31	(14.3)	
							
Tumor grade							0.739
G1 (%)	15	(2.2)	9	(2.0)	6	(2.8)	
G2 (%)	562	(85.5)	377	(85.7)	185	(85.2)	
G3 (%)	60	(9.1)	42	(9.6)	18	(8.3)	
							
UICC							
Stage IA (%) T1N0	21	(3.2)	9	(2.0)	12	(5.5)	0.0003
Stage IB (%) T2N0	73	(11.1)	44	(10.0)	29	(13.4)	
Stage IIA (%) T3N0	202	(30.7)	123	(30.0)	79	(36.4)	
Stage IIB-C (%) T4N0	30	(4.6)	25	(5.7)	5	(2.3)	
Stage III (%) N+	296	(45.1)	220	(50.0)	76	(35.0)	
							
Tumor border configuration							
Infiltrative (%)	418	(63.6)	285	(64.8)	133	(61.3)	0.568
Pushing (%)	218	(33.2)	143	(32.5)	75	(34.6)	
							
Vascular invasion							
No (%)	462	(70.3)	300	(68.2)	162	(74.7)	0.044
Yes (%)	175	(26.6)	129	(29.3)	46	(21.2)	
							
Microsatellite Stability							
Proficient (%)	552	(84.0)	362	(82.3)	190	(87.6)	0.104
Deficient (%)	105	(16.0)	78	(17.7)	27	(12.4)	
							
Rectal cancers (%)	219	(33.3)	131	(29.8)	88	(40.6)	0.013
Rectosigmoid cancers (%)	41	(6.2)	33	(7.5)	8	(3.7)	
							
Median overall survival time (months)	92	0-152	77	0-152	101	0-150	<0.0001
							
5-year overall survival % (95%CI)	55.4	51.6–59.6	49.9	45.2–55.1	66.1	59.9–72.9	0.0003

* Percentages may not add to 100% due to missing values of defined variables.

** Age and tumor size were evaluated using the Mann–Whitney test. Gender, anatomical site, T stage, N stage, grade, vascular invasion, and tumor border configuration were analyzed using the χ2 or Fisher exact test depending on the number of observations. Survival analysis was performed using the Kaplan-Meier method and comparatively analyzed with the log-rank test.

### Prognostic significance of CRC infiltration by OX40/CD134 cells

**Figure 2:**
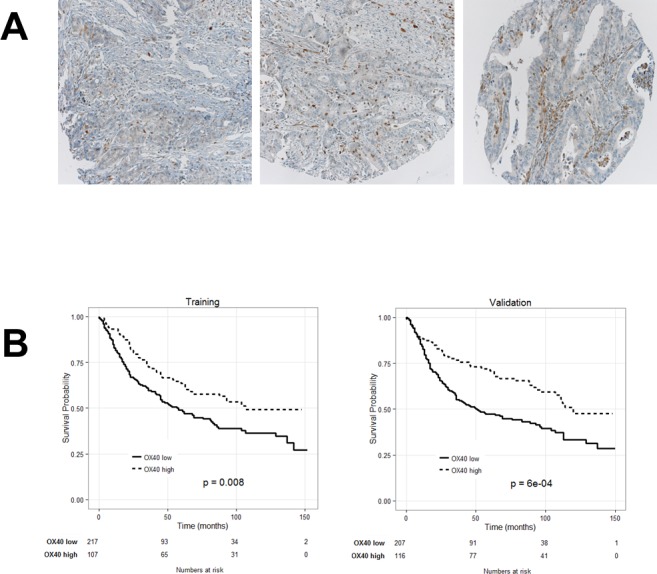
Prognostic significance of OX40 expression by CRC infiltrating cells A. CRC TMA was stained with OX40 specific reagents, as detailed in “materials and methods”. Panel **A.** shows representative punches with different extents of OX40+ cell infiltration (magnification 20X). Kaplan-Meier plots depicting the prognostic significance in randomly generated training and validation cohorts (panel **B.**). Number of events (= deaths) and total number of cases in each cohort are reported.

CRC included in the TMA under investigation were infiltrated to different extents by OX40+ cells (Figure 2A). Clinical-pathological characteristics of training and a validation subset did not significantly differ (supplementary Table 1). Cut-off score of OX40+ CRC infiltrating cells for the assessment of their clinical relevance (*n* = 40) was defined by survival ROC curves in the training set (see above). Table 1 shows data related to each clinical-pathological feature, reported as absolute numbers and percentages. Dropouts due to missing information or to loss of punches during TMA staining and preparation represented < 10% of data.

Kaplan-Meier plots indicate that in both training and validation groups high OX40+ infiltration in CRC is significantly associated with favorable prognosis (Figure 2B). However, the good survival impact of high OX40+ infiltrating cells, failed to reach the threshold of statistical significance in multivariate analysis (*P* = 0.8).

### Synergistic prognostic significance of CRC infiltration by OX40+ and CD8+ cells

OX40 is expressed upon activation by FOXP3+ and CD8+ human T cells [17, 39]. CRC infiltration by cells expressing either marker, known to correlate with favorable clinical course [5, 6, 8, 10, 11], was also associated with favorable prognosis in our cohort of patients (data not shown). Importantly, gene expression data support a significant correlation between OX40 and FOXP3 and CD8 gene expression (see above). Therefore, we explored the potentially synergistic prognostic significance of the expression of these markers.

**Figure 3:**
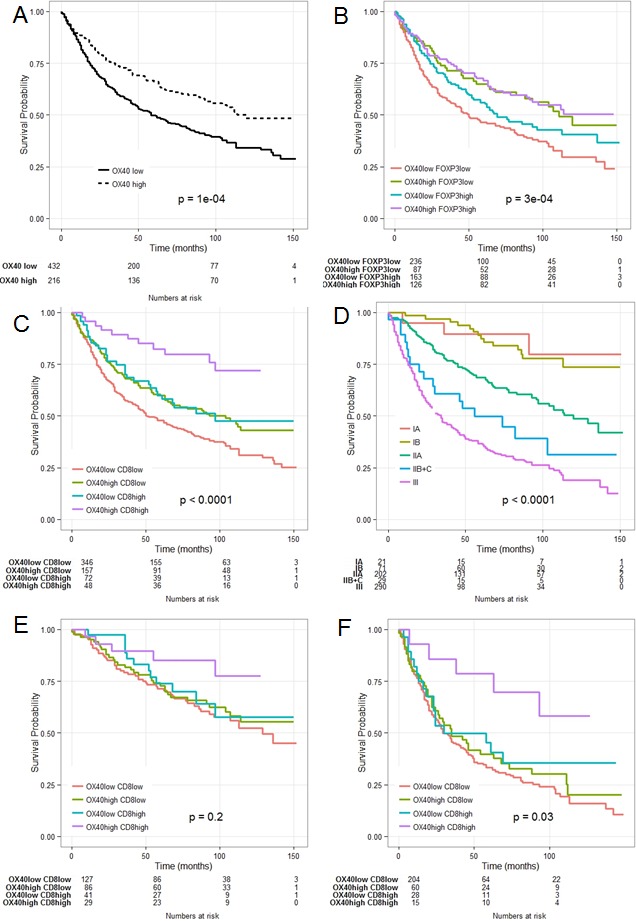
Combined Kaplan- Meier analysis of OX40+ and CD8+ infiltration results in enhanced prognostic significance in CRC OX40+ in the overall collective (panel **A.**); OX40+ combined with FOXP3+ (panel **B.**); OX40+ combined with CD8+ cell infiltration (panel **C.**); comparative evaluation with conventional TNM staging (panel **D.**); OX40+/CD8+ in low stage CRC(panel **E.**) and in high grade (panel **F.**).

Kaplan-Meier plots revealed that combination of high OX40+ and CD8+ infiltration (Figure 3C) was highly significantly associated with increasingly favorable clinical course, as compared to CRC displaying high CD8+ but low OX40+ cell infiltration or CD8+ low but OX40+ high cell infiltration (*P* = 0.0001). In contrast, prognostic significance of OX40+ cell infiltration in CRC was not significantly improved if data were analyzed in combination with FOXP3+ cell infiltration (Figure 3B). However, poor CRC infiltration by OX40+ and FOXP3+ cells was indeed associated with severe prognosis. These findings were confirmed in the “training” and “validation” subsets (data not shown).

Univariate Cox regression analysis of CRC subgroups identified according to high or low OX40+ and/or CD8+ infiltration (supplementary Table 2), revealed that T and N stage and 5 year overall survival rate where associated with OX40+ and CD8+ density. CRC of pT3-4 or pN1-2 stage did show significantly poorer infiltration by OX40+ and CD8+ cells (*P* = 0.00005 and *P* = 0.00004, respectively). Mean survival time for patients bearing tumors with OX40+ and CD8+ high, OX40+ low and CD8+ high, OX40+ high and CD8+ low and OX40+ low and CD8+ low infiltrate was 53.8 (±16.5), 45.7 (±21.4), 43.2 (±21.3) and 38.6 (±22.5) months, respectively. Five year overall survival rate was 82% (CI: 72-94%) for patients presenting with high OX40+ and CD8+ tumor infiltration, and 48% (CI: 43-54%) for patients bearing tumors with poor OX40+ and CD8+ density (*P* = 0.0001).

Multivariate Hazard Cox regression survival analysis revealed that the combination of high density OX40+ and CD8+ cell infiltration (HR = 0.95; 95%CI = 93-97; *P* = 0.006) represents an independent positive prognostic factor for overall survival in CRC. Age (HR = 1.03; 95%CI = 1.01-1.04; *P* < 0.00001), gender (HR = .65; 95%CI = .53-.77; *P* = 0.0003), T-stage (HR = 1.94; 95%CI = 1.82-2.05; *P <* 0.00001), N-stage (HR = 1.88; 95%CI = 1.80-1.97; *P* < 0.00001) and microsatellite instability (HR = 1.82; 95%CI = 1.64-2.01; *P* = 0.001) were also independently associated with favorable prognosis in multivariate survival analysis (Table 2).

**Table 2:** Uni and Multivariate Hazard Cox regression survival analysis

	Univariate			Multivariate		
	HR	95% CI	*p*-values	HR	95% CI	*p*-values
OX40 (continuous)	0.99	0.99-0.99	<0.0001	n.i.		
CD8 (continuous)	0.99	0.98-0.99	0.007	n.i.		
FoxP3 (continuous)	0.88	0.81-0.94	0.049	n.i.		
OX40 ^high/low^	0.63	0.51-0.75	0.0001	n.i.		
CD8 ^high/low^	0.54	0.37-0.70	0.0002	n.i.		
OX40^high^CD8^high^	0.93	0.92-0.95	<0.0001	0.95	0.93-0.97	0.006
FoxP3 ^high/low^	0.77	0.66-0.88	0.02	0.84	0.72-096	0.15
Age	1.02	1.01-1.03	0.0001	1.03	1.02-1.04	<0.00001
Gender (men vs women)	0.67	0.67-0.67	0.0002	0.65	0.53-0.77	0.0003
pT stage	2.25	2.16-2.34	<0.000001	1.94	1.82-2.05	<0.00001
Tumor grade	1.51	1.36–1.67	0.008	1.22	1.03-1.41	0.28
pN stage	2.28	2.21-2.35	<0.000001	1.88	1.80-1.97	<0.00001
Vascular invasion	2.46	2.35-2.58	<0.000001	1.48	1.35-1.62	0.004
Tumor border configuration	2.03	1.90-2.16	<0.000001	1.39	1.24-1.54	0.029
Microsatellite stability (deficient vs. proficient)	1.55	1.38-1.72	0.01	1.82	1.64-2.01	0.001

Uni- and multivariate Cox-regression analyses showing Hazard Ratios and P-values.

The multivariate model included 556 patients, due to missing values related to CD8+ and FOXP3+ density, age, sex, tumor size, tumor grade, vascular invasion, tumor border configuration and microsatellite stability.

n.i.: not included in the final multivariate model.

### Comparative analysis of the prognostic significance of OX40+ and CD8+ infiltration and AJCC staging

Previous studies suggest that infiltration by immune cells might outperform conventional tumor staging in CRC prognostic assessment (4). Therefore, we comparatively evaluated the prognostic significance of OX40+ and CD8+ infiltration and AJCC staging (Figure 3 panel C, D). Survival probability at five years was 85.2% (C.I. 72.6%-99.9%) for patients with low (IA, IB and IIA) AJCC stage and 78.6% (C.I. 59.8%-100%) for patients with high stage (IIB, IIC and III) (Figure 3, panel E, F). Prognostic impact of OX40/CD8 high density infiltration did not reach significance threshold (*P* = 0.2) for patients with low stages, since the overall survival in this cohort is inherently good and analysis of a larger number of patients would be required. However, of peculiar clinical importance is the significant (*P* = 0.03) favorable prognostic impact of high density OX40+ and CD8+ cell infiltration in high AJCC stage CRC. Thus, irrespective of their AJCC staging, CRC highly infiltrated by OX40+ and CD8+ cells are characterized by a prognosis similar to that of stage I patients CRC (Figure 3, panel C-F).

## DISCUSSION

Experimental models show that signaling through OX40 co-stimulatory molecule promotes the generation of T cell memory thereby significantly enhancing antigen specific re-call responses [22, 40]. Furthermore, administration of agonistic OX40 specific mAbs enhances anti-tumor immune responses “*in vivo*” in different models [41]. Responsiveness to treatment is accompanied by increasing densities of OX40+ CD8+ T cells within the tumor tissue and decreasing FOXP3+ Treg infiltration [24]. Based on this background, similar reagents are currently being tested in clinical immunotherapy trials [23, 42].

Although analysis of CRC immune-contexture is increasingly gaining clinical relevance [4-6], prognostic significance of OX40 expression in CRC infiltrating cells has only been explored in one study evaluating a cohort of 72 patients [25].

Here, we report that in CRC OX40 gene expression is significantly correlated to that of CD8, FOXP3, TBET and IRF1 genes, typically expressed in tumors with favorable prognosis [5, 6].

Indeed, the analysis of >600 clinically annotated CRC specimens indicates that OX40+ cell infiltration is significantly associated with increased overall survival, although this favorable prognostic effect could not be confirmed in multivariate analysis.

Most importantly however, a combined evaluation shows for the first time that CRC infiltration by high density OX40+ and CD8+ cells is highly significantly associated with favorable clinical course, as also evident upon multivariate analysis. Strikingly, CRC with high OX40+ and CD8+ cell infiltration, irrespective of their TNM stage, are characterized by a prognosis similar to that of low (IA-IB) stage cancers within the whole cohort under investigation.

In contrast, interestingly, no such effects were observed upon combined analysis of both OX40+ and FOXP3+ cell CRC infiltration.

These data do not provide obvious mechanistic insights. However, it has been shown in “*in vivo*” models that effects of OX40 triggering are enhanced in the presence of “danger” signals [22]. Indeed, CRC carcinogenesis is typically characterized by early loss of the barrier function of intestinal mucosa [43]. Thus, a variety of TLR agonists might provide adequate “danger” signals potentially supporting the induction of antitumor effects associated with OX40 stimulation.

Besides activated T lymphocytes, OX40 expression has also been observed in natural killer T cells and neutrophils [17]. However, in our gene expression studies we did not observe a significant correlation between expression of OX40 and expression of CD16 gene, typically detectable in these cell types.

Our study has limitations, including its retrospective nature. However, data emerging from large retrospective analyses may help in the development of targeted prospective studies, currently being planned by our groups. Furthermore, the cohort investigated in this study includes patients bearing CRC surgically treated between 1985 and 1998, e.g. prior to a widespread use of neoadjuvant treatment regimens. Therefore, although our results may not be fully representative of current clinical treatment, they are more likely to faithfully mirror CRC immunobiology, in the absence of chemo-irradiation treatments.

On the other hand, TMA technology may insufficiently represent tumor tissue heterogeneity. However, punches included in the TMA under investigation are derived from the tumor center and do include at least 50% cancer cells. Furthermore, the number of CRC considered (>600) is likely to compensate at least in part for the diversity of immune contexture within different areas of individual biopsies.

Nevertheless, our data indicate that OX40 and CD8 specific staining may outperform TNM staging, thus potentially contributing to clinical decision making in sizeable groups of patients. On the other hand, they underline the critical relevance of OX40 in CRC immunobiology. Further research is warranted to unravel underlying molecular mechanisms.

## MATERIALS AND METHODS

### Gene expression analysis

Total cellular RNA was extracted from surgical specimens of CRC and autologous healthy mucosa (HM) sampled at distance from the tumor and reverse transcribed [26]. Pre-developed Taqman^®^ assays (Applied Biosystems) were used to quantitatively evaluate the expression of a panel of cytokine and chemokine genes by using ABI Prism 7300 PCR system (Applied Biosystems). Data are reported as relative expression normalized to GAPDH house-keeping gene amplification. Expression of individual genes was analyzed by using the 2^−ΔΔc^_T_ method [27].

### Public databases

Gene expression databases included in Oncomine databank [28] were used to analyze OX40 gene expression in CRC in comparison with normal tissues. Seven databases, including a total of 812 samples, were identified. Skrzypczak [29], Hong [30] and Kaiser [31] databases utilized Human Genome U133 Plus 2.0 technology (Affymetrix), whereas Gaedcke [32] used Agilent platform. Instead, TCGA data were obtained by using next generation sequencing (NGS) technology and Ki [33] data are based on a not pre-defined platform.

### Tissue microarray construction

657 unselected, non-consecutive, clinically annotated, primary CRC specimens were included in the TMA following approval by the Regional Ethical Committee (EKBB, Basel Stadt and Basel Land). Formalin-fixed, paraffin-embedded tissue blocks were prepared according to standard procedures. Tissue cylinders with a diameter of 0.6 mm were punched from morphologically representative areas of each donor block and brought into one recipient paraffin block (30×25mm), using a semi-automated tissue arrayer. Each punch was made from the center of the tumor so that each TMA spot consisted of at least 50% tumor cells.

### Clinical-pathological features

Clinical-pathological data for the patients included in the TMA are listed in Table 1. Briefly, data were collected retrospectively in a non-stratified and non-matched manner. Annotation included patient age, tumor diameter, location, pT/pN stage, grade, histologic subtype, vascular invasion, border configuration, presence of peritumoral lymphocytic inflammation at the invasive tumor front and disease-specific survival. Tumor border configuration and peritumoral lymphocytic inflammation were evaluated using the original H&E slides of the resection specimens corresponding to each tissue microarray punch [34].

### Immunohistochemistry

Standard indirect immunoperoxidase procedures were used for immunohistochemistry (IHC; ABC-Elite, Vector Laboratories, Burlingame, CA). Slides were dewaxed and rehydrated in distilled water. Endogenous peroxidase activity was blocked using 0.5% H2O2. Sections were incubated with 10% normal goat serum (DakoCytomation, Carpinteria, CA) for 20 min and incubated with primary antibody at room temperature. Primary antibodies used were specific for OX40 (polyclonal anti-CD134/OX40, ab119904, Abcam, Cambridge, UK), CD8 (clone C8/144B, DakoCytomation, Switzerland) and FOXP3 (clone 236A/E7, Abcam, Cambridge, UK) [7, 10]. Subsequently, sections were incubated with peroxidase-labelled secondary antibody (DakoCytomation) for 30 min at room temperature. To visualize the antigen, sections were immersed in 3-amino-9-ethylcarbazole plus substrate-chromogen (DakoCytomation) for 30 min, and counterstained with Gill's hematoxylin.

### Evaluation of immunohistochemistry

Immunohistochemical readings were performed by trained research fellows [B.W. or R.D.] and data were independently validated by an additional investigator [L.To.]. Tumor infiltrating cells were counted for each punch (approximately one high power [20x] field). Data regarding CRC infiltration by FOXP3+ and CD8+ were available from our previous publications [7, 8, 10].

### Statistical analysis

Data were analyzed using the Statistical Package Software R (Version 3.1.3, www.r-project.org). Following confirmation histogram and the Kolgomorov-Smirnov test, descriptive statistic included mean ± standard deviation for parameters with Gaussian distribution or percentage of frequencies for occurrences.

The TMA collective of 657 CRC was randomly split into training and validation subsets with approximately equal numbers of patients (*n* = 329 and *n* = 328, respectively). Associations with survival were explored using the Cox proportional hazards regression model. Cut-off values used to classify CRC with low or high immune cell infiltration were obtained by ROC curves (survival ROC package), evaluating sensitivity and false positive rate for the discrimination of survivors and non-survivors with respect to the Kaplan-Meier method, on the training subset and validated on the validation subset [35, 36]. The threshold value for OX40+ infiltration, calculated in the training test was 40 cells/TMA-punch. This value was reconfirmed in the validation set. Further specific scores were set at 10 cells/TMA-punch for CD8 and 17 cells/TMA-punch for FOXP3, as previously calculated in larger collectives by our team [8, 10].

Chi-Square, Fisher's Exact, and Kruskal-Wallis tests were used to determine the association of OX40+ and CD8+ infiltration and clinical-pathological features. Univariate survival analysis was performed by the Kaplan-Meier method and log rank test. The assumption of proportional hazards was verified for all markers by analyzing correlation of Schoenfeld residuals and ranks of individual failure times. Any missing clinical-pathological information was assumed to be missing at random. Subsequently, OX40, CD8, and FOXP3 cell infiltration data were entered into multivariate Cox regression analysis and hazard ratios (HR) and 95% confidence intervals (CI) were used to determine prognostic effects on survival time. *P*-values < 0.05 were considered statistically significant.

## SUPPLEMENTARY MATERIAL TABLES


