# Supplementary material for: OX40 expression enhances the prognostic significance of CD8 positive lymphocyte infiltration in colorectal cancer
Source: Oncotarget. 2015 Sep 29;6(35):37588–99. doi: 10.18632/oncotarget.5940 (PMC4741950; doi:10.18632/oncotarget.5940)
Supplement: Supplementary file 1 [file oncotarget-06-37588-s001.pdf]

OX40 expression enhances the prognostic significance of CD8 positive lymphocyte infiltration in colorectal cancer

Supplementary Material

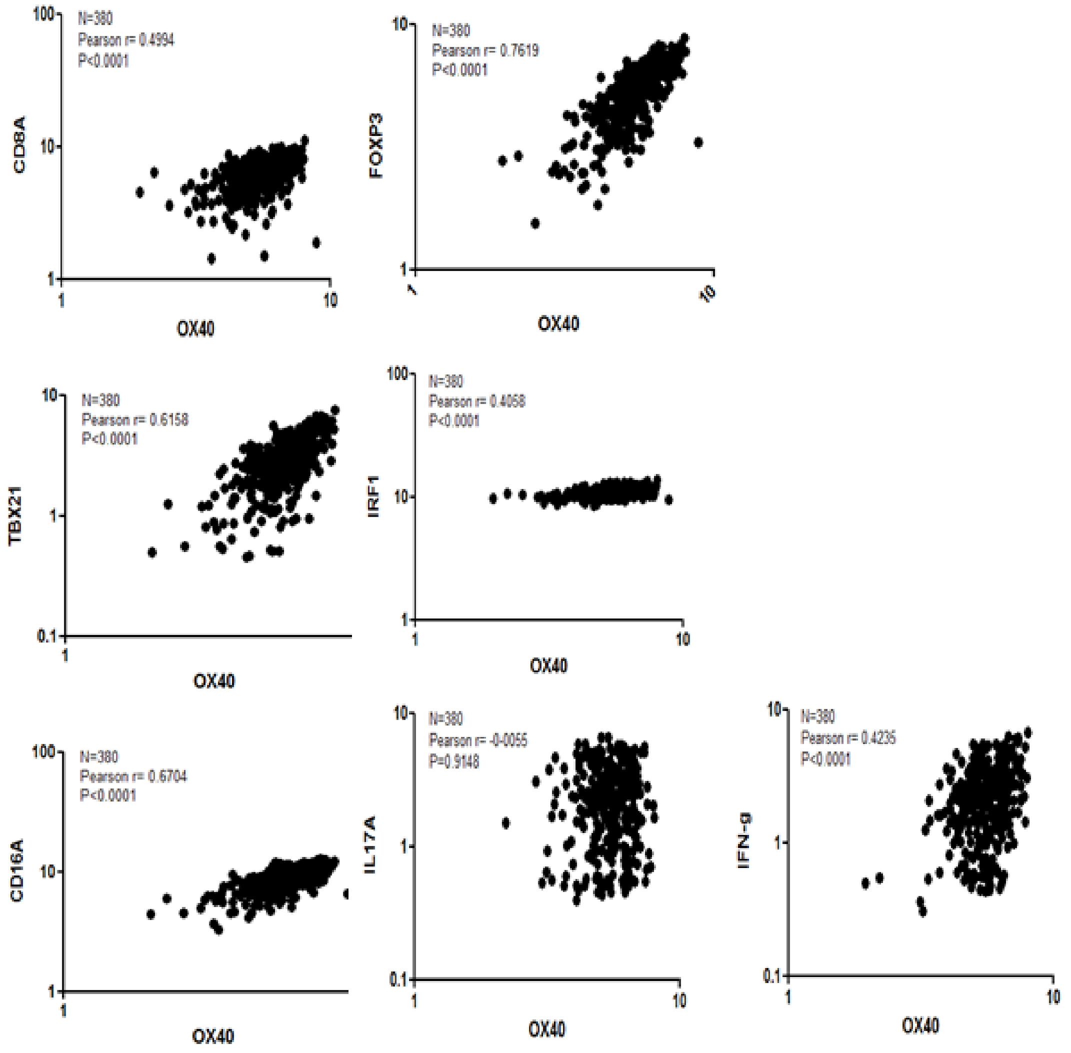

Supplementary figure 1. CRC gene expression profiles from TCGA public database. The expression of the indicated genes was evaluated by next generation sequencing (NGS) in 380 CRC specimens.

Table 1s Comparison of clinical-pathological characteristics of the training and validation subsets (n=328 and n=329)*

	Training		Validation		
Characteristics	N or median, mean	(% or range)	N or median, mean	(% or range)	*P*
Age, years (median, mean)	70, 68.9	(36-90)	72, 70.8	(40-96)	0.041
Tumor size in mm (median, mean)	50, 50.4	(5-170)	50, 51.7	(6-150)	0.593
Gender					0.326
Female (%)	186	(56.7)	173	(52.6)	
Male (%)	142	(43.3)	156	(47.4)	
Anatomic site of the tumor					0.563
Left-sided (%)	217	(66.2)	208	(63.2)	
Right-sided (%)	110	(33.5)	118	(35.9)	
T stage					0.452
T1 (%)	16	(4.8)	13	(4.0)	
T2 (%)	55	(16.8)	48	(14.6)	
T3 (%)	208	(63.4)	208	(63.2)	
T4 (%)	39	(11.9)	52	(15.8)	
N stage					0.9324
N0 (%)	165	(50.3)	168	(51.1)	
N1 (%)	86	(26.2)	88	(26.7)	
N2 (%)	61	(18.6)	67	(20.4)	
Tumor grade					<0.001
G1 (%)	8	(2.4)	7	(2.1)	
G2 (%)	277	(84.5)	285	(86.6)	
G3 (%)	3	(1.0)	30	(9.1)	
UICC					0.053
Stage IA (%) T1N0	12	(3.6)	9	(2.7)	
Stage IB (%) T2N0	39	(11.9)	34	(10.3)	
Stage IIA (%) T3N0	103	(31.4)	99	(30.1)	
Stage IIB-C (%) T4N0	7	(2.1)	23	(7.0)	
Stage III (%) N+	145	(44.2)	151	(45.9)	
Tumor border configuration					0.157
Infiltrative (%)	216	(65.9)	202	(61.4)	
Pushing (%)	99	(30.2)	119	(36.2)	
Vascular invasion					0.717
No (%)	231	(70.4)	231	(70.2)	

**Yes (%)**	**84**	**(25.6)**	**91**	**(27.7)**	
**Microsatellite Stability**					**0.929**
**Proficient (%)**	**276**	**(84.1)**	**276**	**(83.9)**	
**Deficient (%)**	**52**	**(15.9)**	**53**	**(16.1)**	
**Localisation**					**0.379**
**Rectal cancers (%)**	**105**	**(32.0)**	**114**	**(34.7)**	
**Rectosigmoid cancers (%)**	**16**	**(4.9)**	**25**	**(7.6)**	
**Median overall survival time (months)**	**91.5, 83**	**0-152**	**93, 85.8**	**0-151**	**0.469**
**5-year overall survival % (95%CI)**	**58.9**	**53.6 – 64.8**	**52.1**	**46.7 – 58.0**	**0.071**

*Percentages may not add to 100% due to missing values of defined variables. Age and tumor size were evaluated using the Kruskal-Wallis test. Gender, anatomical site, T stage, N stage, grade, vascular invasion, and tumor border configuration were analyzed using the χ^2 test. Survival analysis was performed by using the Kaplan-Meier method.

Supplementary table 2s. Association of OX40+ and CD8+ low and high density infiltration and clinical-pathological features in CRC

		OX40^{high}/ CD8^{high}		OX40^{low}/ CD8^{high}		OX40^{high}/ CD8^{low}		OX40^{low}/ CD8^{low}		p-value
		N=48	(%)	N=73	(%)	N=158	(%)	N=354	(%)	
**Age**	years, mean	68 ± 10		70 ± 10		69 ± 11		71±11		0.10
**Tumor diameter**	mm, mean ± SD	56,7 ± 17,1		55,3 ± 29.2		47,7 ±23.1		51,5 ± 20.8		0.26
**Gender**	Female<br>Male	30<br>18	63<br>37	49<br>24	67<br>33	75<br>82	47<br>52	191<br>162	54<br>46	0.14
**Tumor location**	Left-sided<br>Right-sided	32<br>15	63<br>31	35<br>38	48<br>52	112<br>45	71<br>28	225<br>125	64<br>35	0.007
**Histologic subtype**	Mucinous<br>Non-mucinous	1<br>47	2<br>98	6<br>67	8<br>92	7<br>151	4<br>96	33<br>321	9<br>91	0.11
**pT stage**	pT1-2<br>pT3-4	12<br>33	25<br>69	22<br>48	30<br>66	43<br>108	27<br>68	47<br>300	13<br>85	**0.00005**
**pN stage**	pN0<br>pN1-2	32<br>14	67<br>29	46<br>27	63<br>37	93<br>58	59<br>37	151<br>190	43<br>54	0.00004
**Tumour grade**	G1-2<br>G3	39<br>6	81<br>13	59<br>10	81<br>14	141<br>11	89<br>7	314<br>31	89<br>9	0.29
**Vascular invasion**	Absent<br>Present	35<br>10	73<br>21	52<br>17	71<br>23	120<br>31	76<br>21	234<br>112	66<br>32	0.034
**Tumor border**	Pushing<br>Infiltrating	19<br>26	40<br>54	34<br>35	47<br>48	50<br>101	32<br>64	103<br>242	29<br>68	0.01
**PTL inflammation**	Absent<br>Present	29<br>16	60<br>33	47<br>22	64<br>30	121<br>31	77<br>20	283<br>63	80<br>18	0.007
**Microsatellite stability**	Deficient<br>Proficient	10<br>38	21<br>79	14<br>59	19<br>81	17<br>140	11<br>89	60<br>293	17<br>83	0.189
**5-year overall survival rate**	(95%CI) nr. of deaths	0.82	0.72-0.94	0.60	0.49-0.73	0.59	0.53-0.68	0.48	0.43-0.54	**0.0001**

*Percentages may not add to 100% due to missing values of defined variables. Variables are indicated as absolute numbers, %, median or range. Age and tumor size were evaluated using the Kruskal-Wallis test. Gender, anatomical site, T stage, N stage, grade, vascular invasion, and tumour border configuration were analyzed using the χ^2 test. Survival analysis was evaluated using the Kaplan-Meier method.
